# Comparison of clinical characteristics and disease outcome of COVID-19 and seasonal influenza

**DOI:** 10.1038/s41598-021-85081-0

**Published:** 2021-03-11

**Authors:** Thomas Theo Brehm, Marc van der Meirschen, Annette Hennigs, Kevin Roedl, Dominik Jarczak, Dominic Wichmann, Daniel Frings, Axel Nierhaus, Tim Oqueka, Walter Fiedler, Maximilian Christopeit, Christian Kraef, Alexander Schultze, Marc Lütgehetmann, Marylyn M Addo, Stefan Schmiedel, Stefan Kluge, Julian Schulze zur Wiesch

**Affiliations:** 1 I. Department of Internal Medicine, University Medical Center Hamburg-Eppendorf, Martinistraße 52, 20246 Hamburg, Germany; 2 German Center for Infection Research (DZIF), Partner Site Hamburg-Lübeck-Borstel-Riems, Hamburg, Germany; 3 Department of Intensive Care Medicine, University Medical Center Hamburg-Eppendorf, Martinistraße 52, 20246 Hamburg, Germany; 4 Department of Oncology, Hematology and Bone Marrow Transplantation with Section Pneumology, University Medical Center Hamburg-Eppendorf, Martinistraße 52, 20246 Hamburg, Germany; 5 Department of Stem Cell Transplantation, University Medical Center Hamburg-Eppendorf, 20246 Hamburg, Germany; 6 CHIP (Centre of Excellence for Health, Immunity and Infections), Department of Infectious Disease, Rigshospitalet, University of Copenhagen, Copenhagen, Denmark; 7 Department of Emergency Medicine, University Medical Center Hamburg-Eppendorf, Martinistraße 52, 20246 Hamburg, Germany; 8 Institute of Medical Microbiology, Virology and Hygiene, University Medical Center Hamburg-Eppendorf, Martinistraße 52, 20246 Hamburg, Germany

**Keywords:** Viral infection, Influenza virus

## Abstract

While several studies have described the clinical course of patients with coronavirus disease 2019 (COVID-19), direct comparisons with patients with seasonal influenza are scarce. We compared 166 patients with COVID-19 diagnosed between February 27 and June 14, 2020, and 255 patients with seasonal influenza diagnosed during the 2017–18 season at the same hospital to describe common features and differences in clinical characteristics and course of disease. Patients with COVID-19 were younger (median age [IQR], 59 [45–71] vs 66 [52–77]; P < 0001) and had fewer comorbidities at baseline with a lower mean overall age-adjusted Charlson Comorbidity Index (mean [SD], 3.0 [2.6] vs 4.0 [2.7]; P < 0.001) than patients with seasonal influenza. COVID-19 patients had a longer duration of hospitalization (mean [SD], 25.9 days [26.6 days] vs 17.2 days [21.0 days]; P = 0.002), a more frequent need for oxygen therapy (101 [60.8%] vs 103 [40.4%]; P < 0.001) and invasive ventilation (52 [31.3%] vs 32 [12.5%]; P < 0.001) and were more frequently admitted to the intensive care unit (70 [42.2%] vs 51 [20.0%]; P < 0.001) than seasonal influenza patients. Among immunocompromised patients, those in the COVID-19 group had a higher hospital mortality compared to those in the seasonal influenza group (13 [33.3%] vs 8 [11.6%], P = 0.01). In conclusion, we show that COVID-19 patients were younger and had fewer baseline comorbidities than seasonal influenza patients but were at increased risk for severe illness. The high mortality observed in immunocompromised COVID-19 patients emphasizes the importance of protecting these patient groups from SARS-CoV-2 infection.

## Introduction

On March 11, 2020, the WHO declared the coronavirus disease 2019 (COVID-19), caused by the severe acute respiratory syndrome coronavirus 2 (SARS-CoV-2), a pandemic^1^. While the strain that COVID-19 places on healthcare systems, economies, and societies worldwide is unprecedented, comparisons have often been drawn with the 1918 influenza pandemic^2–4^. Also, the pandemic preparedness plans currently used in many countries are largely based on the experience of several influenza pandemics during the last decades^5^. Thus, a detailed understanding of common features and differences of patients with SARS-CoV-2 and influenza virus infections in the hospital setting can help to plan resources in this ongoing outbreak. COVID-19 and seasonal influenza are viral respiratory infections that are primarily transmitted from person-to-person via respiratory droplets or aerosols among close contacts^6–8^. The clinical presentation of both infections is highly variable and ranges from only mildly symptomatic cases to acute respiratory distress syndrome (ARDS) and death^9^. However, there remains substantial uncertainty regarding the differences between COVID-19 and seasonal influenza with respect to high-risk-populations, clinical course, and case-fatality rates. Most previous studies describing the clinical course of COVID-19 lack a control group^10–13^, and data comparing patients with SARS-CoV-2 infection to patients with other viral respiratory infections such as seasonal influenza in the hospital setting are limited^14–19^. This single-center observational study aimed to systematically compare demographics, clinical characteristics, and disease outcomes of two non-selected cohorts of consecutive patients with SARS-CoV-2 infection and influenza virus infection, respectively, who were all treated at the University Medical Center Hamburg-Eppendorf.

## Materials and methods

### Inclusion criteria

We conducted a retrospective observational study at the University Medical Center Hamburg-Eppendorf to compare two patient cohorts:

i. all adult patients with SARS-CoV-2 infection confirmed by RT-PCR between February 27 and June 14, 2020, treated at the University Medical Center Hamburg-Eppendorf.
ii. all adult patients with seasonal influenza virus infections confirmed by RT-PCR treated at the University Medical Center Hamburg-Eppendorf between December 25, 2017, and April 8, 2018, which is the definition of the influenza season by the Robert Koch Institute, Germany's national public health institute^20,21^.
  The study was reviewed and approved by the Ethics Committee of the Medical Council of Hamburg (WF-017/18 and WF-052/20). Informed consent was waived by the same ethics committee since only anonymous data were analyzed and published. All experiments were performed in accordance with relevant guidelines and regulations. We did not include psychiatric patients and patients younger than 18 years in the study. Also, employees who were diagnosed with COVID-19 by our institution’s screening algorithm but did not require hospitalization were not included in the study^22^.

### Virological diagnosis

According to our hospital standards, all patients admitted with fever or respiratory symptoms were screened for influenza infection by RT-PCR using either the GeneXpert Xpress System (Cepheid, Sunnyvale, USA) or a laboratory-developed assay^23^ throughout the influenza season. After the first case of COVID-19 was confirmed in Germany in January 2020^24^, all symptomatic patients were additionally screened for SARS-CoV-2 infection by RT-PCR using either a laboratory developed test for the NeuMoDx 96 system (NeuMoDx inc., Ann Arbor, USA; distributed by QIAGEN)^25^, a Cobas6800 IVD (Roche Diagnostics, Basel Switzerland), a GeneXpert Xpress System (Cepheid, Sunnyvale, USA) or a Cobas6800-based UCT (Ann Arbor, USA; distributed by QIAGEN)^26^. Only patients with influenza or SARS-CoV-2 infection confirmed by RT-PCR were included in this analysis.

### Clinical data

Clinical information was obtained by retrospective chart review using a standardized case report form, including demographic information, medical history, clinical characteristics, and patient outcome. Detailed information on patients with seasonal influenza treated at our center during the 2017–18 influenza season have been previously published elsewhere^21^. For the current study, COVID-19 and influenza patients were stratified according to whether they were treated as outpatients in our emergency department, were admitted to regular wards of our hospital, or had to be admitted to the intensive care unit (ICU). The age-adjusted Charlson Comorbidity Index (ACCI) was applied to assess comorbidities^27,28^. Patients were classified as immunocompromised if they were allogeneic stem cell transplant recipients, had a hematopoietic malignancy known to cause immune dysfunction or if they received systemic immunosuppressive therapy, chemotherapy, or immunomodulatory agents (see Supplementary Table S1). All clinical outcomes are presented for patients diagnosed with COVID-19 before June 14. Only patients admitted to the regular ward or the ICU, but not patients solely treated as outpatients at the emergency department were included in the subsequent analysis of mean length of hospital stay. At the study endpoint on August 20, two COVID-19 patients with the need for mechanical ventilation were still hospitalized and were thus not included in the analysis of mortality and mean length of hospital stay. Ventilation was defined as the need for high-flow nasal cannula (HFNC), non-invasive ventilation (NIV), or invasive ventilation. The most invasive ventilation method necessary during the hospital stay was used to assign patients to the respective group (e.g., patients that had initially been treated with NIV but subsequently required invasive ventilation were assigned to the latter category).

Antiviral treatment was defined as administration of a specific antiviral agent against influenza and COVID-19, respectively. Patients who participated in placebo-controlled, double-blind treatment studies were assigned to the treatment group for this analysis. Immunotherapy was defined as administration of convalescent plasma, the anti-IL-6 antibody tocilizumab, or the anti-adrenomedullin antibody adrecizumab^29^, while administration of hydrocortisone as part of the treatment regimen for septic shock or the administration of other immunosuppressive agents for indications other than influenza and COVID-19 respectively during the hospitalization was omitted. Bacterial, viral, or fungal co-infections were assessed by chart review, and only isolates determined by the treating physician to be clinically significant were included in the analysis. According to the modified AspICU algorithm^30^, diagnosis of invasive pulmonary aspergillosis was based on the presence of clinical, radiological, and mycological criteria (positive histopathology, positive *Aspergillus* culture, galactomannan ≥ 1.0 in bronchoalveolar lavage fluid or ≥ 0.5 in serum samples).

### Statistical analyses

Continuous variables were expressed as mean and standard deviation (SD) or median and interquartile range (IQR) and compared with Student’s t-test. Categorical variables were expressed as number (%) and compared by Fisher's exact test. A log-rank test was used to compare the proportion of patients discharged alive during the first 90 days of hospitalization. P values less than 0.05 were considered statistically significant. Figures were designed using GraphPad Prism version 8 for macOS (GraphPad Software, La Jolla, California, USA). All other analyses were performed using SPSS, version 21.0 (IBM Corp., Armonk, New York, USA).

## Results

### Demographic information, comorbidities, and immunosuppression

Between February 27 and June 14, 2020, a total of 166 patients with SARS-CoV-2 infection were treated at the University Medical Center Hamburg-Eppendorf. During the 2017–18 influenza season, 255 patients with laboratory-confirmed seasonal influenza were treated at our center^21^. The baseline characteristics of both groups are listed in Table 1. Patients were either seen as outpatients in our emergency department and subsequently hospitalized or discharged, were transferred to our tertiary care center from other hospitals, or contracted nosocomial infections.

**Table 1:** Demographic information, comorbidities, immunosuppression, and immunodeficiency of patients with COVID-19 and seasonal influenza.

	COVID-19 (n = 166)	Influenza (n = 255)	P value
Female, no. (%)	55 (33.1)	111 (43.5)	0.03
Age, median (IQR)	59 (45; 71)	66 (52; 77)	< 0.001
**Comorbidities**			
ACCI, mean (SD)	3.0 (2.6)	4.0 (2.7)	< 0.001
Hypertension, no. (%)	66 (39.8)	131 (51.4)	0.02
Cardiovascular disease, no. (%)	27 (16.3)	73 (28.6)	0.003
Cerebrovascular disease, no. (%)	12 (7.2)	32 (12.5)	0.10
Chronic respiratory disease, no. (%)	25 (15.1)	69 (27.1)	0.004
Chronic liver disease, no. (%)	4 (2.4)	18 (7.1)	0.04
Chronic renal disease, no. (%)	13 (7.8)	48 (18.2)	0.002
Diabetes mellitus, no. (%)	32 (19.3)	52 (20.4)	0.80
SOT recipients, no. (%)	4 (2.4)	23 (9.0)	0.007
**Immunocompromised host**			
Total, no. (%)	39 (23.5)	69 (27.1)	0.43
Corticosteroids, no. (%)	13 (7.8)	40 (58.0)	0.02
CNI/mTORI, no. (%)	6 (3.6)	29 (42.0)	0.006
MTX, no. (%)	2 (1.2)	1 (1.4)	0.57
CD20 antibodies, no. (%)	9 (5.4)	2 (2.9)	0.008
Chemotherapy, no. (%)	20 (12.0)	11 (15.9)	0.004
Acute leukemia, no (%)	13 (7.8)	6 (8.7)	0.01
Lymphoma, no. (%)	8 (4.8)	10 (14.5)	0.81
Allogeneic HCT, no. (%)	5 (3.0)	7 (10.1)	1.00

*COVID-19* coronavirus disease 2019, *IQR* interquartile range, *ACCI* age-adjusted Charlson Comorbidity Index, *SD* standard deviation, *SOT* solid organ transplant, *CNI* calcineurin inhibitor, *mTORI* mTor-inhibitor, *MTX* methotrexate.

**Figure 1:**
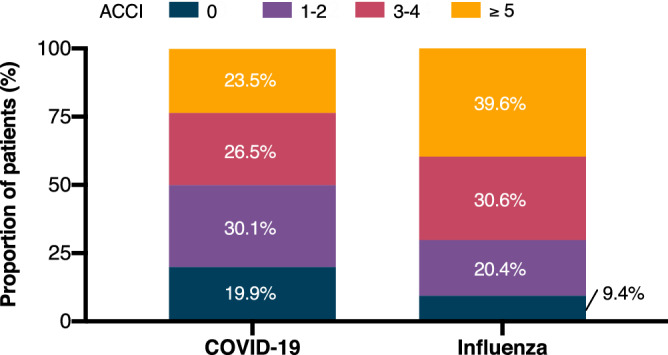
Age-adjusted Charlson comorbidity index (ACCI) of patients with COVID-19 and seasonal influenza. *COVID-19* coronavirus disease 2019, *ACCI* age-adjusted Charlson comorbidity index.

Among COVID-19 patients, the proportion of men was higher than in influenza patients (111 [66.9%] vs 144 [56.5%]; P = 0.03). Patients with SARS-CoV-2 infection were younger (median age [IQR], 59 [45–71] vs 66 [52–77]; P < 0.001) and had a lower overall ACCI (mean [SD], 3.0 [2.6] vs 4.0 [2.7]; P < 0.001) than patients with seasonal influenza (Fig. 1). COVID-19 patients also suffered significantly less frequently from arterial hypertension, cardiovascular disease, chronic respiratory disease, chronic liver disease, chronic renal disease, and were less frequently solid organ transplant recipients compared to influenza patients. The overall proportion of immunocompromised individuals did not differ between the two groups (39 [23.5%] vs 69 [27.1%]; P = 0.43), but COVID-19 patients significantly more often received chemotherapy (20 [12.0%] vs 11 [4.3%]; P = 0.004), CD20 antibodies (9 [5.4%] vs 2 [0.8%]; P = 0.008) or suffered from acute leukemia (13 [7.8%] vs 6 [2.4%]; P = 0.01), while influenza patients were more often treated with systemic corticosteroids (13 [7.8%] vs 40 [15.7%]; P = 0.04) or calcineurin and mTor-inhibitors (6 [3.6%] vs 29 [11.4%]; P = 0.006).

### Hospital course

The hospital course of COVID-19 and seasonal influenza patients is presented in Table 2. No statistically significant differences in the rate of nosocomial infections was observed between the two groups (24 [14.5%] vs 27 [10.6%]; P = 0.29). The rate of patients who were transferred to our center from other hospitals was slightly higher in the COVID-19 group than in the seasonal influenza group (27 [16.3] vs 24 [9.4]; P = 0.046). Of those, all COVID-19 patients and 18 (75%) influenza patients were admitted to the ICU. Ten COVID-19 patients and nine seasonal influenza patients were transferred to our center to be treated with ECMO. Compared with patients with influenza infections, COVID-19 patients were significantly less often treated as outpatients (19 [11.4%] vs 76 [29.8%]; P < 0.001), were more often admitted to the ICU (70 [42.2%] vs 51 [20.0%]; P < 0.001), and had a longer duration of hospitalization (mean [SD], 25.9 days [26.6 days] vs 17.2 days [21.0 days]; P = 0.002). More COVID-19 patients needed some form of oxygen therapy compared to influenza patients (101 [60.8%] vs 103 [40.4%]; P < 0.001). While the rate of patients receiving only low-flow oxygen did not significantly differ between the subgroups, more COVID-19 patients required any form of ventilation (HFNC, NIV, or invasive ventilation) (60 [36.1%] vs 46 [18.0%]; P < 0.001). Patients with SARS-CoV-2 infection more often received HFNC (8 [4.8%] vs 2 [0.8%]; P = 0.02), whereas more influenza patients received NIV (0 [0%] vs 12 [4.7]; P = 0.004). Overall, significantly more COVID-19 patients required tracheal intubation compared with influenza patients (52 [31.3%] vs 32 [12.5%]; P < 0.001).

**Table 2:** Course of disease in patients with COVID-19 and seasonal influenza.

	COVID-19 (n = 166)	Influenza (n = 255)	P value
Mean length of stay—days (SD)^a^	25.9 (26.6)	17.2 (21.0)	0.002
Nosocomial infections, no. (%)	24 (14.5)	27 (10.6)	0.29
Transferrals, no. (%)	27 (16.3)	24 (9.4)	0.046
Outpatient treatment, no. (%)	19 (11.4)	76 (29.8)	< 0.001
Admission to ICU, no. (%)	70 (42.2)	51 (20.0)	< 0.001
**Oxygen therapy**			
Total, no. (%)	101 (60.8)	103 (40.4)	< 0.001
Low-flow oxygen, no. (%)	41 (24.7)	57 (22.4)	0.64
Ventilation (total), no. (%)	60 (36.1)	46 (18.0)	< 0.001
Ventilation (HFNC), no. (%)	8 (4.8)	2 (0.8)	0.02
Ventilation (NIV), no. (%)	0 (0)	12 (4.7)	0.004
Ventilation (Invasive), no. (%)	52 (31.3)	32 (12.5)	< 0.001
Vasopressor therapy, no. (%)	56 (33.7)	36 (14.1)	< 0.001
Renal replacement therapy, no. (%)	35 (21.1)	25 (9.8)	0.002
ECMO, no. (%)	12 (7.2)	11 (4.3)	0.27
Antibiotic treatment, no. (%)	172 (67.5)	112 (67.5)	0.83
**Antiviral treatment**			
Total, no. (%)	27 (16.3)	62 (24.3)	0.05
Oseltamivir, no. (%)		62 (24.3)	
Lopinavir/ritonavir, no. (%)	9 (5.4)		
Remdesivir, no. (%)	9 (5.4)		
Hydroxychloroquine, no. (%)	8 (4.8)		
**Immunotherapy**			
Total, no. (%)	15 (9.0)		
Convalescent plasma, no. (%)	4 (2.4)		
Tocilizumab, no. (%)	3 (1.8)		
Adrecizumab, no. (%)	8 (4.8)		
Deceased, no. (%)	26 (15.9)	23 (9.0)	0.04

*COVID-19* coronavirus disease 2019, *SD* standard deviation, *ICU* intensive care unit, *ECMO* extracorporeal membrane oxygenation, *HFNC* high-flow nasal oxygen, *NIV* non-invasive ventilation.

^a^Only hospitalized COVID-19 and influenza patients were included in the analysis on length of stay. Two COVID-19 patients with the need for mechanical ventilation were still hospitalized and were thus not included in the analysis of mortality and mean length of hospital stay.

COVID-19 patients were more often treated with vasopressors (56 [33.7%] vs 36 [14.1%]; P < 0.001) and renal replacement therapy (56 [21.1%] vs 25 [9.8%]; P = 0.002) than influenza patients. The rate of patients receiving extracorporeal membrane oxygenation (ECMO) did not significantly differ between the two groups. A total of 27 (16.3%) patients with COVID-19 and 62 (24.3%) patients with seasonal influenza received specific antiviral therapy (P = 0.05). In addition, 15 (9.0%) patients in the COVID-19 group received immunotherapy with convalescent plasma, tocilizumab, or adrecizumab. There was no statistically significant difference between the two groups in the rate of patients receiving antibiotic therapy during the hospitalization (112 [67.5%] vs 172 [67.5%]; P = 0.83) (see Supplementary Table S2). However, patients with SARS-CoV-2 infection were less frequently treated with carbapenems (40 [15.9%] vs 57 [34.4%]; P < 0.001) and glycopeptides (25 [9.8%] vs 37 [22.3%]; P = 0.001), but more often received acylaminopenicillins (50 [30.1%] vs 48 [18.8%]; P = 0.009).

The rate of viral (7 [4.2%] vs 11 [4.3%]; P = 1.0), bacterial (18 [10.8%] vs 28 [11.0%]; P = 1.0) and fungal (11 [6.6%] vs 11 [4.3%]; P = 0.37) co-infections was similar in both groups and also the overall spectrum of pathogens did not significantly differ between COVID-19 patients and influenza patients (see Supplementary Table S3). The majority of patients diagnosed with invasive pulmonary aspergillosis did not suffer from underlying immunocompromising conditions in the COVID-19 group (83%, n = 5) nor in the influenza group (62%, n = 10), and mortality was 50% among patients diagnosed with *Aspergillus* co-infections in both groups*.*

The overall hospital mortality was higher in COVID-19 patients compared with influenza patients (26 [15.9%] vs 23 [9.0%]; P = 0.04). Among those patients admitted to the ICU, no differences in mortality were observed between the two groups (23 [32.9%] vs 21 [41.2%]; P = 0.44). Among all immunocompromised patients, those in the COVID-19 group had a higher mortality compared to those in the seasonal influenza group (13 [35.1%] vs 8 [11.0%], P = 0.01). Among patients with SARS-CoV-2 infection, mortality was significantly increased in immunocompromised compared to immunocompetent individuals (13 [35.1%] vs 13 [10.2%]; P = 0.001). In contrast, no significant difference in mortality was observed between immunocompromised and immunocompetent patients with seasonal influenza (8 [11.0%] vs 15 [8.2%]; P = 0.46).

**Figure 2:**
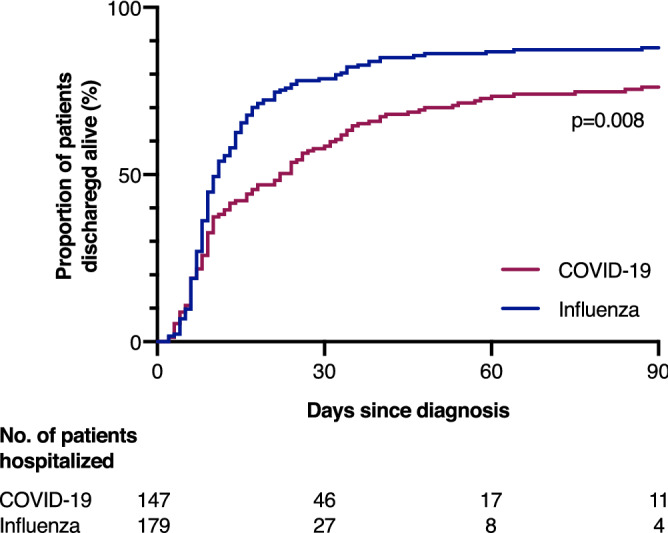
Proportion of hospitalized patients discharged alive during the first 90 days after diagnosis compared by log-rank test.

Notably, all five allogeneic stem cell transplant recipients in the COVID-19 group and one in the seasonal influenza group (14.3%) died (see Supplementary Table S4). Among patients suffering from acute leukemia, hospital mortality was 23.1% (n = 3) in the COVID-19 group and 16.7% (n = 1) in the seasonal influenza group and among patients with lymphomas was 25.0% (n = 2) in the COVID-19 group and 30.0% (n = 3) in the seasonal influenza group. Also, when only comparing immunocompetent patients, those in the COVID-19 group were younger (median age [IQR], 58 years [45–71 years] vs 69 years [56–78 years]; P < 0.001), had a lower ACCI (mean [SD], 2.5 [2.4] vs 4.1 [2.7]; P < 0.001) but had higher rates of hospitalization (89 [82.4%] vs 55 [45.8%]; P < 0.001), a more frequent need for oxygen therapy (71 [55.9%] vs 73 [40.1%]; P = 0.008) and invasive ventilation (33 [26.0%] vs 23 [12.6%]; P = 0.004), more frequent admissions to the intensive care unit (44 [34.6%] vs 35 [19.2%]; P = 0.003). Duration of hospitalization (mean [SD], 19.3 days [19.9 days] vs 14.6 days [15.2 days]; P = 0.05) and hospital mortality (13 [10.2%] vs 15 [8.2%]; P = 0.55) did not significantly differ between immunocompetent COVID-19 and influenza patients. Among all hospitalized patients, the cumulative proportion of those discharged alive during the first 90 days after diagnosis was significantly lower in COVID-19 patients compared to influenza patients (P = 0.008 by log-rank test) (Fig. 2).

Among patients with seasonal influenza, 79 patients were infected with influenza A virus (IAV) and 176 were infected with influenza B virus (IBV). While the hospital mortality did not differ between patients with IAV and IBV infections (9 [11.4%] vs 14 [8.0%,]; P = 0.08), the rate of patients requiring mechanical ventilation was significantly higher in patients with IAV virus infections (14.2%; n = 25/176; P < 0.001) compared to patients with IBV infections (21 [26.6%] vs 25 [14.2%]; P < 0.001).

### Comparison between outpatients, patients admitted to regular wards and patients treated at the ICU

We further compared demographic information, comorbidities, and immune status of patients treated as outpatients, those admitted to regular wards and those admitted to the ICU between the COVID-19 group and the seasonal influenza group (see Supplementary Table S5). Among individuals treated as outpatients, those with COVID-19 were significantly younger (median age [IQR], 41 [31–62] vs 60 [40–70]; P = 0.03) and had a lower ACCI (mean [SD], 1.3 [2.3] vs 2.6 [2.3]; P = 0.04) than those with seasonal influenza. Also, among patients admitted to regular wards, those with COVID-19 were younger (median age [IQR], 55 [44–68] vs 71 [57–79]; P < 0.001) than those with seasonal influenza, had a lower ACCI (mean [SD], 2.4 [2.4] vs 4.8 [2.6]; P < 0.001), and less frequently suffered from hypertension, cardiovascular disease, cerebrovascular disease, chronic respiratory disease, chronic renal disease, diabetes mellitus and were less frequently solid organ transplant recipients. Patients with COVID-19 and patients with seasonal influenza who were admitted to the ICU did not differ in age (median [IQR], 64 years [54–73 years] vs 63 years [55–74 years]; P = 0.59) and ACCI (mean [SD], 4.0 [2.5] vs 4.3 [2.6]; P = 0.63), but patients with COVID-19 less frequently suffered from cardiovascular disease (17 [24.3] vs 22 [43.1%], P = 0.03), chronic respiratory disease (13 [18.6%] vs 19 (37.3%); P = 0.04) and acute leukemia (11 [15.7%] vs 2 [3.9%]; P = 0.04).

## Discussion

In this current study, we directly compared a cohort of 166 consecutive patients with COVID-19 with all 255 patients diagnosed with seasonal influenza during the 2017–18 season treated at the University Medical Center Hamburg-Eppendorf. This comparative approach allowed us to identify common features and differences between those two respiratory infections in the identical hospital setting and provide indications of the strain the COVID-19 pandemic places on hospital resources and capacities.

An important finding to emerge from this comparison is that the majority of COVID-19 and influenza patients were men and that this male predominance was even more pronounced in COVID-19 patients. These observations are consistent with previous studies that have demonstrated a higher overall incidence of seasonal influenza and higher disease severity in male compared to female patients^31,32^. Even though currently available data indicate similar overall COVID-19 case numbers in men and women^33^, male sex has been linked to a generally more severe course of disease with more frequent need for hospitalization and an excess in case fatality rates^34,35^. While the detailed mechanisms underlying those observed sex-differences remain to be established, our study contributes to the growing body of evidence suggesting that male sex is a major risk factor for severe COVID-19^18^.

Another remarkable observation is that patients with SARS-CoV-2 infection were significantly younger than those with seasonal influenza. The median age of COVID-19 patients in our study was lower than in recent multi-center cohort studies of patients hospitalized with SARS-CoV-2 infection in Germany that observed a mean age of 68–70 years^36,37^ and with a recent cohort study analyzing electronic healthcare databases of the US Department of Veterans Affairs that observed a mean age of 69 years^19^. However, our observation that patients with SARS-CoV-2 infection are generally younger than seasonal influenza patients is consistent with previously published data^14,15,18^. Interestingly, among patients that were admitted to the ICU, age did not significantly differ between the two groups. Also, patients requiring ICU admission were significantly older than those treated on regular wards or as outpatients in the COVID-19 group but not in the seasonal influenza group. These findings suggest that age may potentially be a more important driver of disease severity for COVID-19 than for seasonal influenza^19,37^.

Assessing the prevalence of specific comorbidities in COVID-19 patients is paramount to identify and protect patient groups at high risk for severe disease. However, many studies characterizing COVID-19 patients are hampered by the lack of control groups, and the comparability of patient cohorts from different countries or different clinical settings is limited. For example, a large cohort study based on electronic health records and claims data showed that compared to influenza patients, COVID-19 patients had fewer comorbidities in the USA but more comorbidities in South Korea^18^. In our study, COVID-19 patients had a significantly lower ACCI than influenza patients, reflecting a lower overall prevalence of comorbidities. Interestingly, among patients admitted to the ICU, mean ACCI did not differ between the two groups, suggesting that comorbidities may be stronger determinants of disease severity in COVID-19 patients than in seasonal influenza patients. This is further supported by the fact that in the seasonal influenza group, patients admitted to the ICU even had a lower mean ACCI than seasonal influenza patients treated on regular wards. These results may imply that COVID-19 more frequently causes severe disease requiring hospitalization in relatively healthy individuals compared to seasonal influenza but that patients at highest risk for critical disease requiring admission to the ICU are primarily older individuals with comorbidities for both respiratory infections. Remarkably, we observed a significantly lower prevalence of chronic respiratory diseases in COVID-19 patients than influenza patients in the subgroups admitted to regular wards as well in those admitted to the ICU. While underlying respiratory diseases are associated with more severe outcomes of both viral respiratory infections^38,39^, our observations suggest that chronic respiratory conditions may have an even higher impact on the severity of seasonal influenza than on the severity of COVID-19.

Even though COVID-19 patients were generally younger and healthier than influenza patients, the overall course of disease was more severe, with fewer patients treated as outpatients, longer overall duration of hospitalization, more frequent admission to the ICU, more frequent need for oxygen therapy, and invasive ventilation. The overall in-hospital mortality in our cohort was 15.9% in COVID-19 patients and 9.0% in influenza patients. This significantly higher case fatality rate among patients hospitalized with SARS-CoV-2 infection is generally in line with large multi-center observational studies, which have shown an overall in-hospital mortality of 15–22% for COVID-19^10,19,35,40^ and 5–8% for seasonal influenza^19,41,42^. Of note, a number of patients with acute leukemia with SARS-CoV-2 infection experienced a severe clinical course with high hospital mortality and contributed to the relatively high overall mortality in the COVID-19 group. Indeed, while the attributable risk of many immunocompromising conditions and immunosuppressive therapies on severity and outcome of COVID-19 yet needs to be established, there is some evidence that patients with hematological malignancies are at increased risk of severe COVID-19^43–45^. Likewise, there is evidence that immunocompromised individuals have a higher risk for influenza-associated complications^46^. However, no significant effect of immunosuppression and immunodeficiency on overall mortality was observed for influenza patients in our cohort. This might either result from a generally lower severity of immunocompromised state in patients with seasonal influenza compared with COVID-19 patients or reflect a lower impact of immunocompromised state on disease outcome of seasonal influenza compared to COVID-19. Notably, amongst immunocompetent patients, those with COVID-19 had a more severe overall course of disease than those with seasonal influenza, but mortality did not significantly differ between the two groups.

Remarkably, eight patients with SARS-CoV-2 infection were successfully treated with HFNC and did, therefore, not require tracheal intubation. While NIV is associated with an increased risk of aerosolization and nosocomial transmission^47^, HFNC has been suggested to benefit COVID-19 patients with acute hypoxemic respiratory failure without this increased risk of SARS-CoV-2 transmission to patients and healthcare workers^48,49^.

In our cohort, the prevalence of invasive pulmonary aspergillosis was similar among critically ill influenza and COVID-19 patients, even in the absence of underlying immunocompromising conditions and was associated with significant mortality. While invasive pulmonary aspergillosis typically occurs in severely immunocompromised hosts, it has also been shown to be a frequent complication in immunocompetent but critically ill patients with influenza infections^28,50^. More recently, several studies have reported a high prevalence of invasive pulmonary aspergillosis in COVID-19 patients admitted to the ICU^51–54^, suggesting that those patients might be at equally high risk to develop co-infections with *Aspergillus*. Our observations emphasize that clinicians need to be aware of this complication in order to conduct prompt and comprehensive analysis in high-risk patients^54,55^.

While bacterial co-infections in general and the different causative bacterial pathogens, in particular, were equally common in seasonal influenza patients and COVID-19 patients, the latter were significantly less frequently treated with carbapenems and glycopeptides. The reason for this less frequent use of these broad-spectrum antibiotic classes may be a more established antibiotic stewardship program at our hospital in the 2020 outbreak versus the 2017–2018 season.

Our study is subject to a number of limitations. Firstly, patients diagnosed at and referred to our tertiary care center may have generally more baseline comorbidities and a more severe course of disease than patients in different clinical settings, so our results may not apply to all other patient cohorts. Secondly, while COVID-19 and seasonal influenza are both viral respiratory infections that share a common transmission route and cause similar symptoms, they are subject to several important differences that considerably limit the comparability of our cohorts. While most humans have pre-existing immunity to seasonal influenza virus strains and vaccines are available for high-risk populations, the COVID-19 pandemic has demonstrated the potential impact of a novel pathogen on an immunologically naive population. Remarkably, only four of the 23 deceased seasonal influenza patients in our study cohort were vaccinated against seasonal influenza. Thirdly, given relatively low influenza case numbers throughout the 2019–2020 season, we compared COVID-19 patients to patients diagnosed with seasonal influenza throughout the 2017–18 season, when most laboratory-confirmed influenza cases were attributed to influenza B virus, and mismatch between the trivalent vaccine and the circulating strains occurred^21^. Notably, the rate of patients requiring mechanical ventilation was significantly higher in patients with IAV infections compared to patients with IBV infections. These findings demonstrate that extrapolation of disease outcomes related to other influenza seasons should be performed with caution. Fourthly, the screening and treatment algorithm at our center was different for seasonal influenza and COVID-19. While generally only symptomatic patients are tested for influenza infections, all patients admitted to our hospital were screened for SARS-CoV-2 infection from April 20 onwards, which may contribute to sampling bias and result in a higher number of COVID-19 patients with mild or subclinical infections. COVID-19 patients were treated strictly according to a hospital-wide algorithm and, therefore, may, for example, have received low-flow oxygen at higher blood oxygen saturation levels than seasonal influenza patients. Lastly, it is important to note that the significantly lower age and fewer comorbidities in COVID-19 patients compared with influenza patients admitted to regular wards may also, to some extent, reflect a lower threshold for admitting patients with SARS-CoV-2 infection to the hospital due to the challenges of self-quarantine from household members and less data on reliable predictors for disease severity. Likewise, duration of hospitalization in COVID-19 patients may be prolonged due to PCR positivity and not due to clinical necessity, which may place additional strain on healthcare systems.

Despite those limitations, our study can provide some relevant insights into common features and differences in clinical characteristics and required hospital resources between patients with COVID-19 during the early phase of the pandemic and those with seasonal influenza. Prospective multi-center studies using standardized admission, treatment, and outcome protocols are needed to confirm the current findings and to assess the changes in morbidity and mortality of COVID-19 as well as of seasonal influenza throughout this evolving pandemic.

## Conclusion

In conclusion, we demonstrate that patients treated with SARS-CoV-2 infection during the early phase of the pandemic were younger and healthier than those with seasonal influenza infections. However, patients with SARS-CoV-2 infection had a generally more severe course of disease. These results suggest that this phase of the COVID-19 epidemic was associated with a higher demand for both critical care and regular care hospital beds than seasonal influenza epidemics with similar patient numbers. Importantly, the high mortality in COVID-19 patients with hematological malignancies and older patients emphasizes the importance of protecting these patient groups from SARS-CoV-2 infection. While patients hospitalized with SARS-CoV-2 infection in the future will likely differ from those in our cohort in clinical characteristics and disease outcome due to changes in disease epidemiology and novel therapeutic agents and strategies, our observations provide important insights for the future course of the COVID-19 pandemic.

## Supplementary Information

Supplementary Information.
