# Supplementary material for: Comparison of clinical characteristics and disease outcome of COVID-19 and seasonal influenza
Source: Sci Rep. 2021 Mar 11;11:5803. doi: 10.1038/s41598-021-85081-0 (PMC7970952; doi:10.1038/s41598-021-85081-0)
Supplement: Supplementary file 1 — Supplementary Information. [file 41598_2021_85081_MOESM1_ESM.docx]

**Comparison of clinical characteristics and disease outcomes of COVID-19 and seasonal influenza**

Thomas Theo Brehm^1,2^, Marc van der Meirschen^1^, Annette Hennigs^1^, Kevin Roedl^3^, Dominik Jarczak^3^, Dominic Wichmann^3^, Daniel Frings^3^, Axel Nierhaus^3^, Tim Oqueka^4^, Walter Fiedler^4^, Maximilian Christopeit^5^, Christian Kraef^1,6^, Alexander Schultze^7^, Marc Lütgehetmann^2,8^, Marylyn M Addo^1,2^, Stefan Schmiedel^1,2^, Stefan Kluge^3,#^, Julian Schulze zur Wiesch^1,2,#^

**Affiliations**

^1^ I. Department of Internal Medicine, University Medical Center Hamburg-Eppendorf, Martinistraße 52, 20246 Hamburg, Germany

^2^ German Center for Infection Research (DZIF), Partner Site Hamburg-Lübeck-Borstel-Riems

^3^ Department of Intensive Care Medicine, University Medical Center Hamburg-Eppendorf, Martinistraße 52, 20246 Hamburg, Germany

^4^ Department of Oncology, Hematology and Bone Marrow Transplantation with Section Pneumology, University Medical Center Hamburg-Eppendorf, Martinistraße 52, 20249 Hamburg, Germany

^5^ Department of Stem Cell Transplantation, University Medical Center Hamburg-Eppendorf, 20246 Hamburg, Germany

^6^ CHIP (Centre of Excellence for Health, Immunity and Infections), Department of Infectious Disease, Rigshospitalet, University of Copenhagen, Copenhagen, Denmark

^7^ Department of Emergency Medicine, University Medical Center Hamburg-Eppendorf, Martinistraße 52, 20246 Hamburg, Germany

^8^ Institute of Medical Microbiology, Virology and Hygiene, University Medical Center Hamburg-Eppendorf, Martinistraße 52, 20246 Hamburg, Germany

^#^ contributed equally

**Supplementary table S1 –** List of all drugs considered systemic immunosuppressive therapy, chemotherapy, or immunomodulatory agents in the study cohort

| Asparaginase |
| --- |
| Azacitidin |
| Basiliximab |
| Blinatimumab |
| Bortezomib |
| Calcineurin inhibitors |
| CAR-T cells |
| Carboplatin |
| Cisplatin |
| Corticosteroids (≥ 5 mg/day prednisolone-equivalent) |
| Cyclophosphamid |
| Cytarabin |
| Daunorubicin |
| Doxorubicin |
| Etoposid |
| mTor-inhibitors |
| MTX |
| Obinutuzumab |
| Paclitaxel |
| Pembrolizumab |
| Rituximab |
| Ruxolitinub |
| Thalidomid |
| Ventoclax |

**Supplementary table S2 -** Antibiotic treatment of patients with COVID-19 and seasonal influenza

|  | **COVID-19 (n=166)** | **Influenza (n=255)** | **P Value** |
| --- | --- | --- | --- |
| Total, No. (%) | 112 (67.5) | 172 (67.5) | 0.83 |
| Penicillins, No. (%) | 4 (2.4) | 6 (3.6) | 0.20 |
| Aminopenicillins, No. (%) | 70 (27.5) | 56 (33.7) | 0.19 |
| Acylaminopenicillins, No. (%) | 48 (18.8) | 50 (30.1) | 0.009 |
| Cephalosporins, No. (%) | 73 (28.6) | 45 (27.1) | 0.82 |
| Fluorquinolones, No. (%) | 51 (20.0) | 46 (27.7) | 0.08 |
| Macrolides, No. (%) | 38 (14.9) | 27 (16.3) | 0.78 |
| Carbapenems, No. (%) | 40 (15.7) | 57 (34.3) | <0.001 |
| Glycopeptides, No. (%) | 25 (9.8) | 37 (22.3) | 0.001 |
| Linezolid, No. (%) | 18 (7.1) | 8 (4.8) | 0.41 |
| Other, No. (%) | 16 (9.6) | 22 (8.6) | 0.86 |

Abbreviations: COVID-19, coronavirus disease 2019

**Supplementary table S3 -** Bacterial, viral and fungal co-infections in patients with COVID-19 and seasonal influenza

|  | **COVID-19 (n=166)** | **Influenza (n=255)** | **P Value** |
| --- | --- | --- | --- |
| **Bacterial co-infections** |  |  |  |
| Total, No. (%) | 18 (10.8) | 28 (11.0) | 1.0 |
| Place of detection |  |  |  |
| Blood culture, No. (%) | 9 (5.4) | 15 (5.9) | 1.0 |
| Respiratory secretions, No. (%) | 14 (8.4) | 17 (6.7) | 0.57 |
| Bacterial pathogens |  |  |  |
| *Pseudomonas* spp., No. (%) | 2 (1.2) | 6 (2.4) | 0.49 |
| *Enterococcus* spp., No. (%) | 8 (4.8) | 5 (2.0) | 0.15 |
| *Klebsiella* spp., No. (%) | 8 (4.8) | 6 (2.4) | 0.18 |
| *Staphylococcus aureus*, No. (%) | 1 (0.6) | 9 (3.5) | 0.10 |
| *Acinetobacter baumanii*, No. (%) | 3 (1.8) | 2 (0.8) | 0.39 |
| *Streptococcus* spp., No. (%) | 5 (3.0) | 5 (2.0) | 0.53 |
| Other, No. (%) | 3 (1.8) | 5 (2.0) | 1.0 |
| **Viral co-infections** |  |  |  |
| Total, No. (%) | 7 (4.2) | 11 (4.3) | 1.0 |
| Place of detection |  |  |  |
| Respiratory secretions, No. (%) | 7 (4.2) | 11 (4.3) | 1.0 |
| Viral pathogens |  |  |  |
| HSV, No. (%) | 7 (4.2) | 8 (3.1) | 0.60 |
| CMV, No. (%) | 0 (0) | 3 (1.2) | 0.28 |
| **Fungal co-infections** |  |  |  |
| Total, No. (%) | 11 (6.6) | 11 (4.3) | 0.37 |
| Candidemia, No. (%) | 2 (1.2) | 0 (0) | 0.16 |
| Invasive pulmonary aspergillosis, No. (%) | 6 (3.6) | 16 (6.3) | 0.27 |

Abbreviations: COVID-19, coronavirus disease 2019; HSV, herpes simplex virus; CMV, cytomegalovirus

**Supplementary table S4 – Mortality in immunosuppressed patients with COVID-19 and influenza**

|  | **COVID-19 (n=39)** | | | **Influenza (n=69)** | | |
| --- | --- | --- | --- | --- | --- | --- |
|  | **Deceased (n=13)** | **Not deceased (n=26)** | **P value** | **Deceased (n=8)** | **Not deceased (n=65)** | **P value** |
| Corticosteroids, No. (%) | 5 (38.5) | 8 (61.5) | 0.73 | 5 (12.5) | 35 (87.5) | 0.72 |
| CNI/mTORI, No. (%) | 3 (50.0) | 3 (50.0) | 0.38 | 2 (6.9) | 27 (93.1) | 0.47 |
| MTX, No. (%) | 1 (50.0) | 1 (50.0) | 1.0 | 0 (0) | 1 (100) | 1.0 |
| CD20 antibodies, No. (%) | 2 (22.2) | 7 (77.8) | 0.69 | 0 (0) | 2 (100) | 1.0 |
| Chemotherapy, No. (%) | 8 (40.0) | 12 (60.0) | 0.50 | 2 (18.2) | 9 (81.8) | 0.60 |
| Acute leukemia, No (%) | 3 (23.1) | 10 (76.9) | 0.48 | 1 (16.7) | 5 (83.3) | 0.52 |
| Lymphoma, No. (%) | 2 (25.0) | 6 (75.0) | 0.69 | 3 (30.0) | 7 (70.0) | 0.07 |
| Allogeneic HCT, No. (%) | 5 (100) | 0 (0) | 0.002 | 1 (14.3) | 6 (85.7) | 0.57 |

Abbreviations: COVID-19, coronavirus disease 2019; CNI, calcineurin inhibitor; mTORI, mTor-inhibitor; MTX, methotrexate

**Supplementary table S5 -** Demographic information, comorbidities and immunosuppression and immunodeficiency of patients with COVID-19 and seasonal influenza treated as outpatients, on regular wards and on intensive care units

|  | **Outpatients** | | | **Regular ward** | | | **Intensive care unit** | | |
| --- | --- | --- | --- | --- | --- | --- | --- | --- | --- |
|  | **COVID-19 (n=19)** | **Influenza**  **(n=76)** | **P Value** | **COVID-19 (n=77)** | **Influenza (n=128)** | **P Value** | **COVID-19 (n=70)** | **Influenza (n=51)** | **P Value** |
| Female, No. (%) | 8 (42.1) | 39 (51.1) | 0.61 | 29 (37.7) | 51 (39.8) | 0.77 | 18 (25.7) | 22 (43.1) | 0.05 |
| Age, Median (IQR) | 41 (31;62) | 60 (40;70) | 0.03 | 55 (44;68) | 71 (57;79) | <0.001 | 64(54;73) | 63(55;74) | 0.59 |
| Comorbidities |  |  |  |  |  |  |  |  |  |
| ACCI, Mean (SD) | 1.3 (2.3) | 2.6 (2.3) | 0.04 | 2.4 (2.4) | 4.8 (2.6) | <0.001 | 4.00 (2.5) | 4.3 (2.6) | 0.63 |
| Hypertension, No. (%) | 5 (26.3) | 23 (30.3) | 1.0 | 25 (32.5) | 76 (59.4) | <0.001 | 36 (51.4) | 32 (62.7) | 0.27 |
| Cardiovascular disease, No. (%) | 1 (5.3) | 14 (18.4) | 0.29 | 9 (11.7) | 37 (28.9) | 0.005 | 17 (24.3) | 22 (43.1) | 0.03 |
| Cerebrovascular disease, No. (%) | 1 (5.3) | 7 (9.2) | 1.0 | 3 (3.9) | 19 (14.8) | 0.02 | 8 (11.4) | 6 (11.8) | 1.0 |
| Chronic respiratory disease, No. (%) | 3 (15.8) | 18 (23.7) | 0.55 | 9 (11.7) | 32 (25.0) | 0.03 | 13 (18.6) | 19 (37.3) | 0.04 |
| Chronic liver disease, No. (%) | 0 | 3 (3.9) | 1.0 | 2 (2.6) | 11 (8.6) | 0.14 | 2 (2.9) | 4 (7.8) | 0.24 |
| Chronic renal disease, No. (%) | 1 (5.3) | 6 (7.9) | 1.0 | 6 (7.8) | 32 (25.0) | 0.003 | 6 (8.6) | 10 (19.6) | 0.10 |
| Diabetes mellitus, No. (%) | 1 (5.3) | 12 (15.8) | 0.45 | 6 (7.8) | 28 (21.9) | 0.01 | 25 (35.7) | 12 (23.5) | 0.17 |
| SOT recipients, No. (%) | 0 | 3 (3.9) | 1.0 | 2 (2.6) | 17 (13.3) | 0.01 | 2 (2.9) | 3 (5.9) | 0.65 |
| Immunosuppression and immunodeficiency | | | | | | | | | |
| Total, No. (%) | 0 | 11 (14.5) | 0.11 | 13 (16.9) | 42 (32.8) | 0.02 | 26 (37.1) | 16 (31.4) | 0.57 |
| Corticosteroids, No. (%) | 0 | 3 (3.9) | 1.0 | 3 (3.9) | 26 (20.3) | 0.001 | 11 (15.7) | 11 (21.6) | 0.48 |
| CNI/mTORI, No. (%) | 0 | 4 (5.3) | 0.58 | 2 (2.6) | 19 (14.8) | 0.004 | 4 (5.7) | 6 (11.8) | 0.32 |
| MTX, No. (%) | 0 | 0 | - | 0 | 1 (0.8) | 1.0 | 2 (2.9) | 0 | 0.51 |
| CD20 antibodies, No. (%) | 0 | 0 | - | 4 (5.2) | 2 (1.6) | 0.20 | 5 (7.1) | 0 | 0.07 |
| Chemotherapy, No. (%) | 0 | 2 (2.6) | 1.0 | 5 (6.5) | 5 (3.9) | 0.51 | 15 (2.1) | 4 (7.8) | 0.047 |
| Acute leukemia, No (%) | 0 | 2 (2.6) | 1.0 | 3 (3.9) | 3 (2.3) | 0.67 | 10 (14.3) | 2 (3.9) | 0.07 |
| Lymphoma, No. (%) | 0 | 0 | 1.0 | 3 (3.9) | 5 (3.9) | 1.0 | 4 (5.7) | 3 (5.9) | 1.0 |
| Allogeneic HCT, No. (%) | 0 | 2 (2.6) | 1.0 | 1 (1.3) | 2 (1.6) | 1.0 | 4 (5.7) | 3 (5.9) | 1.0 |

Abbreviations: COVID-19, coronavirus disease 2019; IQR, interquartile range; ACCI, age-adjusted Charlson Comorbidity Index; SD, standard deviation; SOT, solid organ transplant; CNI, calcineurin inhibitor; mTORI, mTor-inhibitor, MTX, methotrexate
